# Facilitation of motor excitability during listening to spoken sentences is not modulated by noise or semantic coherence

**DOI:** 10.1016/j.cortex.2018.02.007

**Published:** 2018-06

**Authors:** Muriel T.N. Panouillères, Rowan Boyles, Jennifer Chesters, Kate E. Watkins, Riikka Möttönen

**Affiliations:** a Department of Experimental Psychology, University of Oxford, Oxford, United Kingdom; b School of Psychology, University of Nottingham, Nottingham, United Kingdom

**Keywords:** Articulatory motor cortex, Semantic context, Speech in noise, Speech perception, Transcranial magnetic stimulation

## Abstract

Comprehending speech can be particularly challenging in a noisy environment and in the absence of semantic context. It has been proposed that the articulatory motor system would be recruited especially in difficult listening conditions. However, it remains unknown how signal-to-noise ratio (SNR) and semantic context affect the recruitment of the articulatory motor system when listening to continuous speech. The aim of the present study was to address the hypothesis that involvement of the articulatory motor cortex increases when the intelligibility and clarity of the spoken sentences decreases, because of noise and the lack of semantic context. We applied Transcranial Magnetic Stimulation (TMS) to the lip and hand representations in the primary motor cortex and measured motor evoked potentials from the lip and hand muscles, respectively, to evaluate motor excitability when young adults listened to sentences. In Experiment 1, we found that the excitability of the lip motor cortex was facilitated during listening to both semantically anomalous and coherent sentences in noise relative to non-speech baselines, but neither SNR nor semantic context modulated the facilitation. In Experiment 2, we replicated these findings and found no difference in the excitability of the lip motor cortex between sentences in noise and clear sentences without noise. Thus, our results show that the articulatory motor cortex is involved in speech processing even in optimal and ecologically valid listening conditions and that its involvement is not modulated by the intelligibility and clarity of speech.

## 1 Introduction

Speech perception is a demanding skill that is supported by an extensive brain network. Although the human auditory system is critical for the processing of acoustic speech signals, numerous neuroimaging studies have shown that frontal cortical regions such as inferior frontal gyrus (IFG) and premotor cortex are also activated during speech perception (Adank, 2012, Callan et al., 2010, Hervais-Adelman et al., 2012, Londei et al., 2010, Osnes et al., 2011, Pulvermüller et al., 2006, Skipper et al., 2017, Skipper et al., 2005, Szenkovits et al., 2012, Wilson et al., 2004). Transcranial magnetic stimulation (TMS) combined with electromyography provides a method to measure excitability of the representations of the articulators in the primary motor cortex during speech perception (Adank et al., 2017, Möttönen and Watkins, 2012, Mottonen et al., 2014a). Single TMS pulses over the representations of the articulators in the primary motor cortex elicit motor evoked potentials (MEPs) in the targeted muscles. Changes in the size of MEPs reflect changes in the excitability of the motor pathways connecting the cortical representations with the corresponding muscles. Using this technique, several studies have demonstrated that the excitability of the primary motor cortex, which controls articulatory gestures to produce speech, is enhanced during listening to speech (Fadiga et al., 2002, Murakami et al., 2011, Murakami et al., 2013, Nuttall et al., 2017, Nuttall et al., 2016, Watkins et al., 2003).

It has been proposed that the articulatory motor system is a complementary system, recruited when listening to speech in challenging conditions (Wilson, 2009). Some MEP studies have indeed shown that listening to speech in noise enhances the excitability of the lip motor cortex more than listening to speech (sentences or syllables) without noise (Murakami et al., 2011, Nuttall et al., 2017). These MEP studies did not however include a wide range of noise levels and therefore it is currently unknown how signal-to-noise ratio (SNR) of speech signal affects the excitability of the articulatory motor cortex. Several functional Magnetic Resonance Imaging (fMRI) studies have found an increased activation in the left IFG and premotor cortex to degraded speech compared to clear speech (Adank and Devlin, 2010, Du et al., 2014, Evans and Davis, 2015, Hervais-Adelman et al., 2012, Osnes et al., 2011). It is not however completely clear whether these increased frontal activations are related to increased involvement of the speech motor system in speech processing, increased involvement of additional cognitive processes (Eckert, Teubner-Rhodes, & Vaden, 2016; Peelle, 2018) or motor tasks. Recently, Du et al. (2014) investigated the activation of the motor and auditory systems during a phoneme categorization task at various SNR levels. The activation of the speech motor system (premotor cortex and posterior IFG) correlated negatively with the SNR-modulated accuracy. Furthermore, multi-voxel pattern analyses showed that the speech motor cortex successfully categorized the phonemes at lower SNR levels than the auditory system. These findings support the idea that the speech motor system has a compensatory role when categorizing speech sounds in noisy conditions. However, since the participants performed an active syllable identification task on every trial via a button press using their right hand, it is unclear whether the activations of the left primary motor cortex/pre-motor cortex were related to this task or processing of speech sounds (see for a discussion of this point Schomers & Pulvermüller, 2016). In addition, it remains unknown how SNR affects the activity of the articulatory motor system during passive listening to more natural speech signals such as sentences.

In everyday life, speech comprehension is supported by semantic context as it improves intelligibility of continuous speech in noise (Davis et al., 2005, Miller and Isard, 1963, Obleser et al., 2007). For example, word report scores for semantically coherent sentences like “the coin was thrown onto the floor” are higher than for semantically anomalous sentences like “the boot was grown onto the mouth” across a wide range of SNR levels (Davis, Ford, Kherif, & Johnsrude, 2011). Neuroimaging studies have shown that semantic context affects activity in the IFG and its connectivity with other brain regions (Davis et al., 2005, Davis et al., 2011, Obleser et al., 2007, Sohoglu et al., 2012). These frontal activations are likely to be related to linguistic or semantic processing of the sentences, not speech processing in the articulatory motor cortex. It can be hypothesized that if the involvement of the articulatory motor system increases in challenging conditions, then it should show greater activation when listening to semantically anomalous sentences relative to semantically coherent sentences especially in noise.

In the present study, we aimed to address the hypothesis that the recruitment of the articulatory motor cortex increases when the intelligibility of the spoken sentences decreases and speech perception becomes more challenging. We modulated intelligibility of spoken sentences by manipulating their SNR and semantic coherence. MEPs from the lip and the hand muscles were measured while participants passively listened to semantically coherent and anomalous sentences and non-speech signals in two experiments. The aim of Experiment 1 was to test how a range of five SNR levels affects motor excitability. The aim of Experiment 2 was to test replicability of the results of Experiment 1 and to determine whether motor excitability is sensitive to the presence of noise when processing spoken sentences. Experiment 2 included sentences at two SNR levels and sentences without noise. The comparison between lip and hand MEPs allowed us to test whether listening to speech enhances excitability in the articulatory motor system specifically.

## 2 Materials and methods

### 2.1 Participants

Forty participants were recruited in Experiment 1. The data of eleven participants were excluded because of 1) unreliable motor evoked potentials (MEPs) in the lip muscle (*N* = 4), 2) artefacts in the recording preventing the accurate offline detection of lip MEPs (*N* = 5), 3) lip background muscle contraction (*N* = 1) and 4) proportion of correctly reported words for the anomalous sentences was below 40% at the highest SNR (0 dB) (*N* = 1). In total, we report the data from twenty-nine participants for Experiment 1. Thirteen participants were in the hand group (7 females –age: 24.4 ± 5.3 years old) and sixteen in the lip group (5 females – age: 22.9 ± 3.9 years old).

Thirty-five participants were recruited in Experiment 2. The data of ten participants was excluded based on 1) lip background muscle contraction (*N* = 8), 2) artefacts in the recording preventing the accurate offline detection of lip MEPs (*N* = 1) and 3) the reported clarity on a scale from 1 to 8 in the anomalous sentences at the highest SNR (0 dB) was below 3.2 (equivalent to 40% reported accuracy in Experiment 1; *N* = 1). In total, we report the data from sixteen participants in the hand group (10 females – age: 22.4 ± 2.8 years old) and nine participants in the lip group (six females – age: 24.4 ± 3.6 years old).

The fifty-four participants for whom data is reported in the present study are right-handed, native-English speakers, with no known neurological, psychiatric, hearing or language impairment. All participants gave their written informed consent and were screened prior inclusion for contraindications to TMS. Experimental procedures conformed to the Code of Ethics of the World Medical Association (Declaration of Helsinki) and were approved by Oxfordshire NHS Research Ethics Committee B (REC Reference Number 10/H0605/7).

### 2.2 Electromyography

Electromyography (EMG) activity was recorded using surface electrodes (22 × 30 mm ARBO neonatal electrocardiogram electrodes). Recordings from the right orbicularis oris were taken from electrodes attached to the right upper and lower lip. Recordings from the right first dorsal interosseous muscle were taken from electrodes attached to the belly and tendon of the muscle. The ground electrode was attached to the forehead. The raw EMG signal was amplified (gain: 1000), bandpass filtered (1–1000 Hz) and sampled (5000 Hz) via a CED 1902 four-channel amplifier, a CED 1401 analog-to-digital converter and a computer running Spike2 (Cambridge Electronic Design). The EMG signals were stored on the computer for off-line analysis.

### 2.3 Transcranial Magnetic Stimulation

All TMS pulses were monophasic, generated by Magstim 200 (Magstim, Whitland, UK) and delivered through a 70-mm figure of eight coil. The position of the coil over the left motor cortex was adjusted until a robust motor-evoked potential (MEP) was observed in the contralateral target muscle (either hand or lip). Single-pulse TMS was delivered for every trial to allow recording MEPs from the resting target muscle. The intensity of the stimulation was set in order for an MEP of at least 1 mV peak-to-peak for the hand and at least 0.2 mV for the lip to be consistently produced in the resting muscle for five consecutive TMS pulses. The mean intensity used for the lip groups was of 69.8% (±6.9%) and of 58.2% (±8.2%) in Experiments 1 and 2, respectively. For the hand stimulation, the averaged intensity was of 59.7% (±12.0%) and of 56.5 (±9.5%) in Experiments 1 and 2, respectively.

### 2.4 Stimuli

The stimuli used in the present study have been used in previous fMRI studies (Davis et al., 2011, Rodd et al., 2005). The set comprised 200 declarative sentences between 6 and 13 words in length. One hundred sentences from this set were semantically coherent (e.g., “the coin was thrown onto the floor” – see Appendix A for complete list of coherent sentences). The remaining hundred sentences were semantically anomalous created by randomly substituting content words matched for syntactic class, frequency of occurrence and number of syllables. The anomalous sentences were identical to the normal sentences in terms of phonological, lexical and syntactic properties but lacked coherent meaning (e.g., “the boot was grown onto the mouth” – see Appendix B for complete list of anomalous sentences). All 200 sentences (1.2–3.5 s in duration, speech rate 238 words/minute) were produced by a male speaker of British English and digitized at a sampling rate of 44.1 Khz. The sentences were degraded with noise according to the procedure described in Davis et al. (2011). Speech in noise was generated using Praat software by adding a continuous speech-spectrum noise background to sentences at the various signal-to-noise ratio (SNR: 0 dB, −1 dB, −2 dB, −3 dB, −4 dB). The overall amplitude of each speech-in-noise stimuli was reduced to match the amplitude of the original sentence. Pure Signal Correlated Noise (SCN) stimuli and White Noise (WN) stimuli were also used in the present study. The SCN stimuli were generated by replacing speech signal of sentences with a signal-correlated noise version of the speech. Signal-correlated noise is a waveform with the same spectral profile and amplitude envelope as the original speech but consisting entirely of noise. Sentences processed in this way sound like a rhythmic sequence of noise bursts, carry no linguistic information and are entirely unintelligible. Sound files of a sentence at the different SNRs, of a SCN stimulus and of a WN stimulus are available as supplementary material.

### 2.5 Experimental set-up and procedures

In both experiments, participants were either assigned to the lip or the hand stimulation group. Participants sat in front of a computer presenting the stimuli using Presentation® software (Neurobehavioral Systems, Inc., Berkeley, CA, USA). Audio stimuli were presented to the participants through insert earphones (Etymotic, Elk Grove Village, IL, USA).

Supplementary audio related to this article can be found at https://doi.org/10.1016/j.cortex.2018.02.007.

The following are the supplementary data related to this article:

speech 0dB

speech -1dB

speech -2dB

speech -3dB

speech -4dB

clear speech

SCN

WN

#### 2.5.1 Experiment 1

This experiment included two blocks, one with coherent sentences and the other one with anomalous sentences. In each block, 100 different sentences were presented, 20 of each at the SNR of 0 dB, −1 dB, −2 dB, −3 dB and −4 dB, along with 30 SCN stimuli and 30 WN stimuli. For each block, the order of the 160 stimuli was randomized and the order of the blocks (coherent and anomalous) was counterbalanced across participants. Moreover, the SNR of each sentence was varied across participants. Participants were instructed to listen to the sentences or to the noise while keeping both their lip and hand muscles relaxed. For each stimulus (sentence, SCN or WN), a single-pulse of TMS was delivered to elicit an MEP. For the sentence stimuli, it was delivered 150 ms after the onset of the final content word. This was chosen as a reliable way of matching the point at which TMS was delivered across sentences, as the final content word was likely to be the most predictable. For the WN and SCN stimuli, the pulse was delivered close to the end of the stimuli, matching the timing of the pulses for sentence stimuli. The average inter-pulse-interval was 6s (range: 4.24s–7.94s). Within each block, there was a short break every 32 trials.

After the completion of the two blocks, participants listened to the 100 anomalous and 100 coherent sentences again and repeated them out loud. For each type of sentences, 20 sentences were presented at the 5 SNR (0 dB, −1 dB, −2 dB, −3 dB and −4 dB). The experimenter assessed the accuracy of the participant's response during the task by calculating the number of correctly reported words out of the total number of words in the sentence.

#### 2.5.2 Experiment 2

This experiment included only one session. Subjects were presented with 40 clear speech stimuli, 40 at 0 dB and 40 at −2 dB from the set of sentences described earlier. Half of the sentences were anomalous sentences and half of them were coherent sentences. The experiment included also 30 WN and 30 SCN stimuli. All stimuli were presented in random order. As in Experiment 1, a single TMS pulse was delivered in the beginning of the last word of the sentence (as above) eliciting an MEP in either the relaxed hand or the relaxed lip muscle.

After the TMS session, participants listened to a subset of the sentences again (90 in total, 15 of each SNR for the normal sentences, 15 of each SNR for the anomalous sentences) and were asked, after hearing each sentence, to rate the clarity of the sentence on a scale from 1 to 8, using the computer keyboard.

### 2.6 Behavioural analysis

In Experiment 1, we calculated the proportions of correctly reported words for each SNR level and sentence type in each participant. In Experiment 2, we calculated means of clarity scores for each SNR level and sentence type in each participant.

### 2.7 MEP analysis

MEPs were analysed on a trial-by-trial basis using in-house software written in Matlab (Mathworks Inc, Natick, USA). Maximal and minimal peaks of the MEPs were automatically detected using a fixed window following the TMS pulse: [15–40 ms] for the hand and [12–35 ms] for the lip. The detection was checked manually by the experimenter. The absolute value of the background muscle activity was averaged across the 100 ms preceding the TMS pulse and trials with a mean absolute value of background muscle activity higher than 2 standard deviations of the average for each TMS session were excluded. Outliers MEPs with values above or below 2 standard deviations of the mean for each experimental condition (sentence types and SNR levels) were removed. Based on these criteria, 6.45 ± 2.2% and 5.87 ± 2.1% of the total number of trials were excluded from Experiment 1 and Experiment 2, respectively. After removing outliers, we calculated MEP z-scores for each experimental condition relative to the WN in each participant.

### 2.8 Statistical analysis

Statistical analyses were performed with the SPSS Statistics software package (IBM, Armonk NY, USA). The proportion of correctly reported words, the perceived clarity of the sentences and the normalised MEP z-scores were analysed using separate ANOVAs with the within-subject factors semantic coherence (coherent *vs* anomalous) and SNR (Experiment 1: 0 dB, −1 dB, −2 dB, −3 dB, −4 dB; Experiment 2: clear; 0 dB, −2 dB) and the between-subjects factor group (hand *vs* lip). In both experiments, because of a lack of SNR effect and semantic effect and interaction involving these factors, the MEP z-scores were averaged across SNR levels and semantic types. In Experiments 1 and 2, these averaged z-scores were submitted to an ANOVA with the within-subject factor stimulus (speech *vs* SCN) and the between-subjects factor group (hand *vs* lip). Finally, we run a 3-way ANOVA with the between-subject factors TMS group (hand *vs* lip) and experiment (1 *vs* 2) and the within-subject factor stimulus (coherent *vs* anomalous *vs* SCN) using the average MEP z-scores to test for differences between experiments. This was followed by two separate ANOVAs for the lip and hand groups with the between-subject factor experiment and the within-subject factor stimulus.

For all ANOVAs, Greenhouse–Geisser corrections to the degrees of freedom were applied if Mauchly's sphericity test revealed a violation of the assumption of sphericity for any of the factors in the ANOVAs. Significance level was set at *p* < .05.

## 3 Results

### 3.1 Experiment 1

The aim of Experiment 1 was to examine whether and to what extent SNR and semantic coherence of sentences modulate motor excitability. We measured MEPs from the lip and hand muscles while participants passively listened to sentences. After MEP measurements, we tested how SNR and semantic coherence affected the intelligibility of the sentences using a behavioural word-report task.

#### 3.1.1 Effect of semantic coherence and SNR on intelligibility of spoken sentences

**Fig. 1:**
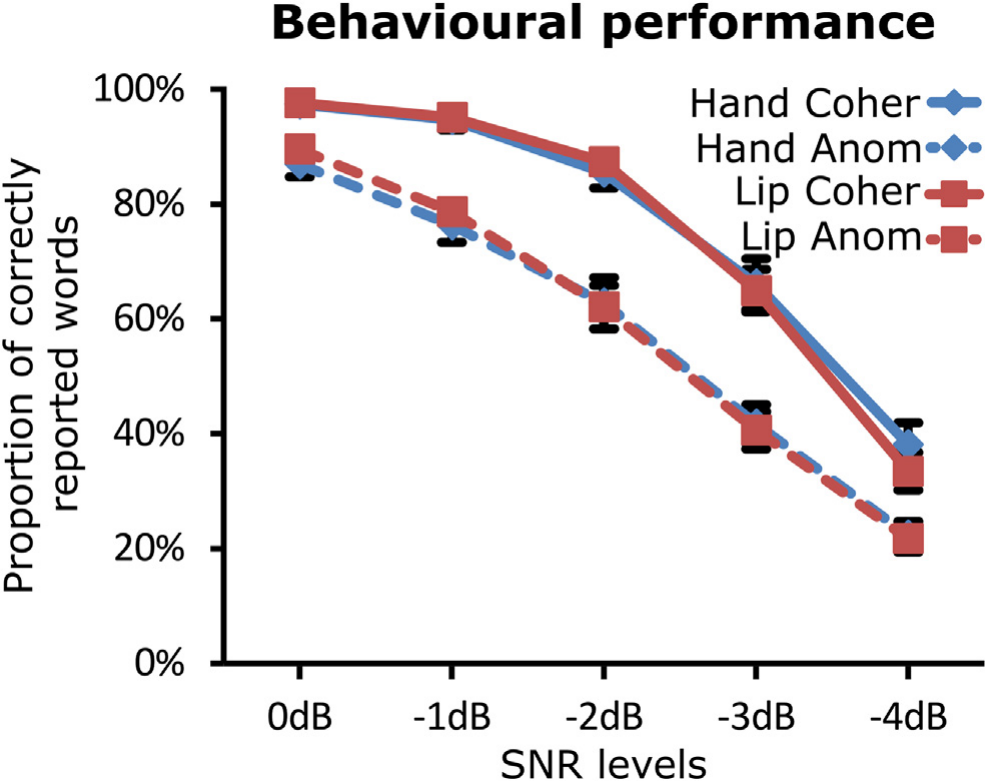
Effects of SNR levels and sentence coherence on word-report accuracy in Experiment 1. The proportion of correctly reported words is represented as a function of the SNR levels for the hand (blue diamonds) and lip (red squares) groups, separately for the coherent (Coher: continuous lines) and anomalous (Anom: dashed lines) sentences. Error bars are standard error of the mean.

Fig. 1 presents the proportions of correctly reported words for the semantically coherent and anomalous sentences at the various SNRs (0 dB, −1 dB, −2 dB, −3 dB, −4 dB). The proportion of correctly reported words decreased with the SNR [main effect of SNR: F(2,67) = 581.73, *p* < .001]. Moreover, the intelligibility of the anomalous sentences was lower than intelligibility of the coherent sentences [main effect of semantic coherence: F(1,27) = 141.31, *p* < .001]. An interaction between the semantic coherence and SNR was also significant [F(2,67) = 18.01, *p* < .001], mostly because the difference between anomalous and coherent sentences was greater at the intermediate SNRs (−1 to −3 dB) than at other SNRs. No significant main effect of TMS group and interactions involving this factor were found (all *p*-values greater than .55). Note that the sentences were not completely unintelligible at the highest level of noise (−4 dB), as the number of correctly reported words in this condition significantly differed from 0 (one sample *t*-test, *p* < .001). In conclusion, SNR levels and semantic coherence modulated intelligibility similarly in the hand and lip groups.

**Fig. 2:**
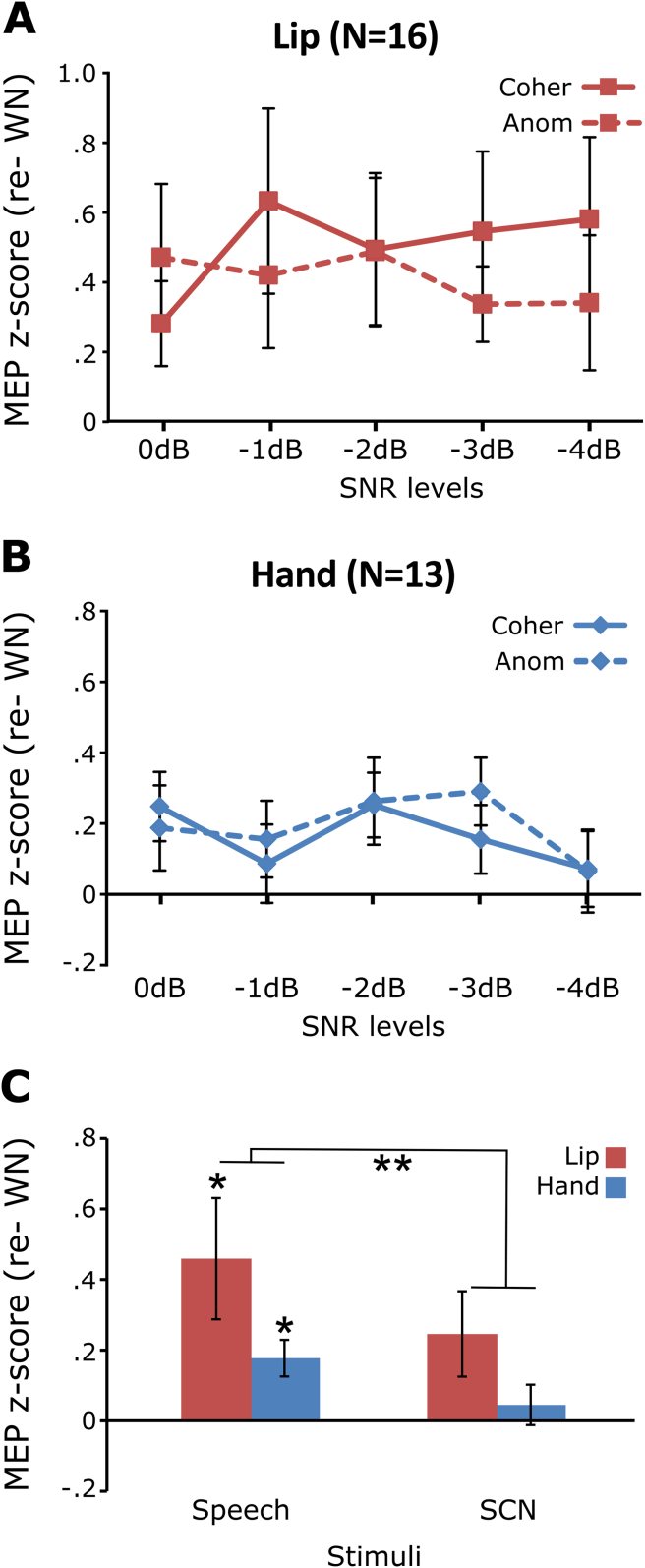
MEP z-scores during the perception of sentences in noise and Signal Correlated Noise (SCN). The MEPs elicited during the perception of sentences in the five SNR levels are represented for the lip (A) and hand group (B), separately for the coherent (Coher) and Anomalous (Anom) sentences. These z-scores are shown averaged for the speech stimuli and compared to the non-speech stimuli (SCN) for the lip (red bars) and hand (blue bars) groups (C). Asterisks above the bars represent significant differences from zero (WN baseline) and asterisks between the bars represent differences between stimuli: *p < .05 and **p < .01. Error bars are standard error of the mean.

#### 3.1.2 Motor excitability when listening to sentences

The MEP z-scores normalised to the WN baseline are presented for anomalous and coherent sentences at the five SNR levels in Fig. 2A and B for the lip and hand groups, respectively. To test whether SNR and semantic affected motor excitability, a three-way ANOVA for the MEP z-scores with SNR and semantic coherence as within-subject factors and group as a between-subjects factor was carried out. There was no significant main effect or interaction involving the SNR factor or the semantic coherence factor (all *p*-values greater than .27), suggesting that motor excitability was stable across the five SNR levels and across the sentence types. The z-scores for all speech stimuli (across five SNR levels and coherent and anomalous sentences) were then averaged for each participant in order to examine whether listening to speech enhanced motor excitability relative to non-speech stimuli.

Fig. 2C presents the MEP z-scores for the speech and SCN stimuli in the lip and hand groups. To assess whether the stimulus type modulated motor excitability, an ANOVA with the within-subject factor stimulus type (speech *vs* SCN) and the between-subjects factor TMS group (hand *vs* lip) was performed. The main effect of the stimulus type was significant (F[1,27] = 16.96, *p* < .001), showing that motor excitability was greater when listening to speech than when listening to SCN. There was no significant main effect of the group factor nor any interaction between group and stimuli (all *p*-values greater than .16).

To assess whether motor excitability was enhanced relative to the WN baseline, the MEP z-scores (normalized relative to WN), were compared statistically to 0. The lip MEP z-scores were significantly greater than 0 for the speech stimuli [one sample *t*-tests: t(15) = 2.67, *p* < .05] and was slightly enhanced for the SCN [one sample *t*-tests: t(15) = 2.03, *p* = .06]. The hand MEP z-scores were greater than 0 only for the speech stimuli [t(12) = 3.42, *p* < .01] but not for the SCN [t(12) = .79, *p* = .45].

In summary, in Experiment 1 we found no modulatory effect of SNR or semantic coherence on motor excitability during listening to spoken sentences. As expected, listening to speech however enhanced the excitability of the lip motor cortex relative to non-speech sounds (WN and SCN). Unexpectedly, the excitability of the hand motor cortex was also enhanced during listening to speech relative to non-speech sounds.

### 3.2 Experiment 2

In Experiment 1, we found no effect of SNR on the motor excitability when participants listened to spoken sentences. However, all sentences were presented in noise. In Experiment 2, we examined whether the presence of noise can enhance motor excitability by using sentences with (SNRs: 0 and −2 dB) and without noise (clear speech). Furthermore, in Experiment 1 we presented anomalous and coherent sentences in different blocks while MEPs were recorded from the lip and hand muscles, which may have reduced the reliability of the comparison between sentences types. In Experiment 2, we presented anomalous and coherent sentences in the same block in order to examine the effect of semantic coherence on motor excitability more reliably. Similarly to Experiment 1, two non-speech stimuli were included in the block (WN baseline and SCN) and participants were either assigned to the hand or to the lip group. After MEP measurements, we tested how the presence of noise and semantic coherence affect the perceived clarity of the spoken sentences using a rating task.

#### 3.2.1 Effect of semantic coherence and SNR on perceived clarity of spoken sentences

**Fig. 3:**
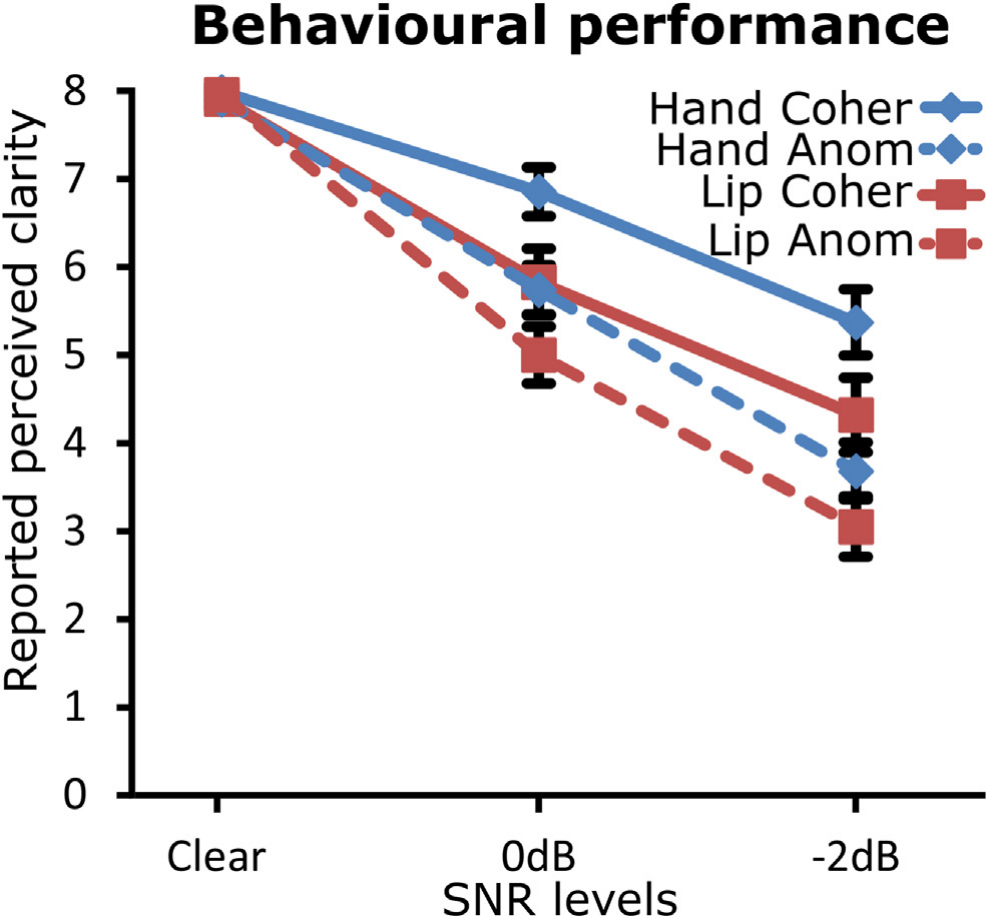
Effects of the presence of noise and sentence coherence on reported perceived clarity in Experiment 2. The perceived clarity is represented as a function of the SNR levels for the hand (blue diamonds) and lip (red squares) groups, separately for the coherent (continuous lines) and anomalous (dashed lines) sentences. Error bars are standard error of the mean.

Fig. 3 presents the mean clarity ratings for the anomalous and coherent sentences presented without noise and with SNRs of 0 dB and −2 dB. Similarly to Experiment 1, the main effects of semantic coherence [F(1,23) = 107.23, *p* < .001] and SNR [F(1, 34) = 157.66, *p* < .001] as well as an interaction between semantic coherence and SNR [F(2,46) = 44.43, *p* < .001] were significant. This demonstrates that the anomalous sentences were less clear than the coherent sentences at the 0 dB and −2 dB SNR levels, whereas there was no difference in clarity in the absence of noise between the two sentences types. There was a non-significant tendency for lower clarity ratings in the lip group than in hand group [F(1,23) = 3.35, *p* = .08], but no significant interactions involving TMS group [semantic coherence × group: F(1,23) = 3.13, *p* = .09; SNR × group: F(1,34) = 2.58, *p* = .10; semantic coherence × SNR × group: F(2,46) = .40, *p* = .67]. In sum, SNR and semantic coherence modulated the perceptual clarity of the sentences in both groups.

**Fig. 4:**
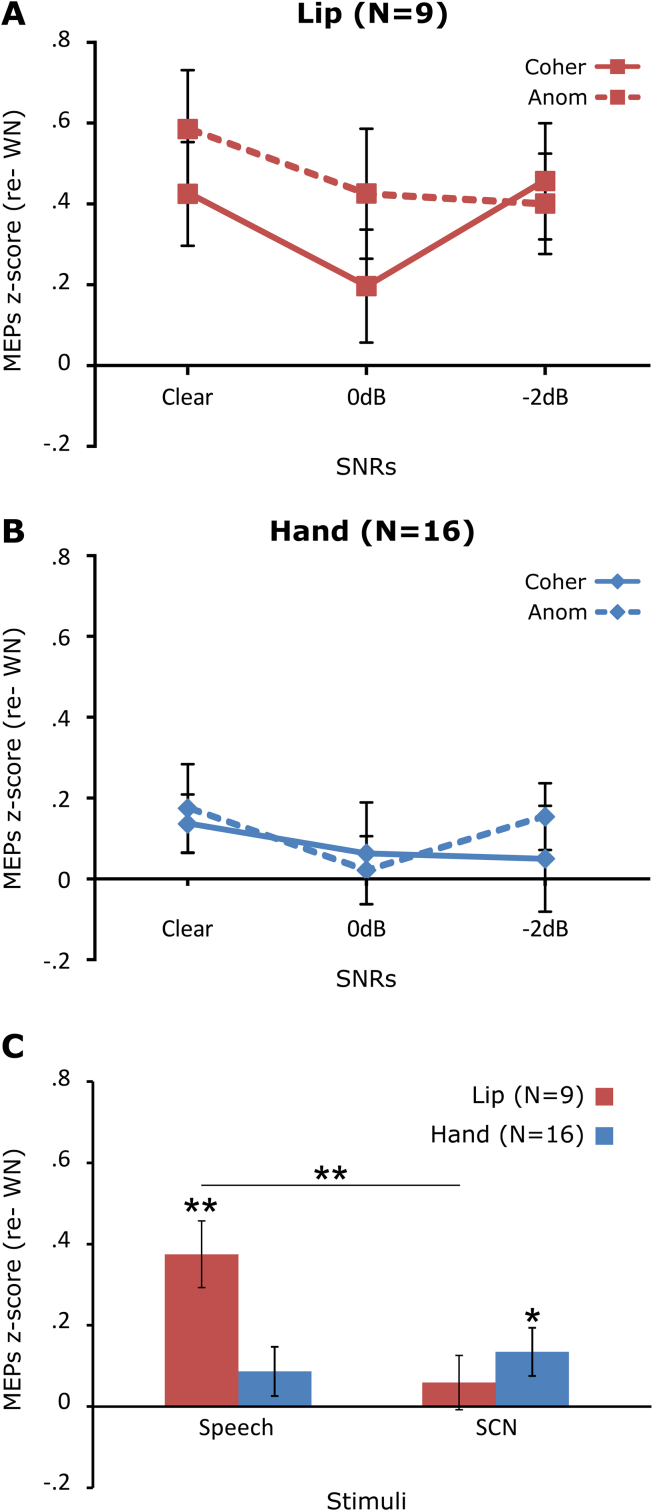
MEP z-scores during the perception of sentences with and without noise and Signal Correlated Noise (SCN). The MEPs elicited during the perception of sentences in clear speech, at 0 dB and −2 dB are represented for the lip (A) and hand group (B), separately for the coherent (Coher) and Anomalous (Anom) sentences. These z-scores are represented averaged across the speech stimuli and for the non-speech stimuli (SCN) for the lip (red bars) and hand (blue bars) groups (C). Asterisks above the bars represent significant differences from zero (WN baseline) and asterisks between the bars represent significant differences between stimuli: *p < .05 and **p < .01. Error bars are standard error of the mean.

#### 3.2.2 Specific facilitation of the lip motor cortex when listening to speech

The MEP z-scores, normalised to the WN baseline, for anomalous and coherent sentences are presented as a function of SNR in Fig. 4A and B for the lip and hand groups, respectively. To evaluate whether the presence of noise and semantic coherence modulates motor excitability during listening to speech, an ANOVA with the within-subject factors SNR (clear *vs* 0 dB *vs* −2 dB) and semantic coherence (coherent *vs* anomalous) and the between-subjects factor TMS group (hand *vs* lip) was carried out. There was no significant main effect of SNR, semantic coherence or interactions involving these factors (all *p*-values greater than .12), suggesting that motor excitability was not modulated by the presence of noise nor by semantic coherence. The main effect of the TMS group was significant [F(1,23) = 3.24, *p* < .05] showing that listening to speech enhanced the excitability of the lip motor cortex more than the excitability of the hand motor cortex. Because the SNR and semantic coherence factors had no effect, we averaged the MEP z-scores across the three SNR levels and the two sentence types for each participant (Fig. 4C). To test whether motor excitability was modulated by stimulus type, an ANOVA with the within-subjects factor stimulus (speech *vs* SCN) and the between-subjects factor group (hand *vs* lip) was carried out on the MEP z-scores. Stimulus type differently modulated the excitability of the lip and hand motor cortex [stimulus effect: F(1,23) = 3.99, *p* = .06; stimulus × group interaction: F(1,23) = 10.47, *p* < .01]. Pairwise comparisons revealed that listening to speech stimuli enhanced the excitability of the lip motor cortex relative to SCN [t(8) = 4.1, *p* < .01] but that the excitability of the hand motor cortex was not modulated by the stimulus type [i.e., speech *vs*. non-speech: t(15) = −.95, *p* = .36].

To assess whether motor excitability was enhanced relative to the WN baseline, the MEP z-scores (normalized relative to WN) were statistically compared to 0. The lip MEP z-scores were significantly greater than 0 for the speech stimuli [one sample *t*-test: t(8) = 4.19, *p* < .01] but not for the SCN [t(8) = 1.04, *p* = .33]. In the hand group, the MEP z-scores for the speech stimuli did not differ from 0 [t(15) = 1.32, *p* = .21], but they were marginally greater than 0 for the SCN [t(15) = 2.12, *p* = .05].

In summary, results of Experiment 2 showed that passive listening to spoken sentences enhances the excitability of the lip motor cortex more than listening to non-speech sounds (SCN and WN), and that neither semantic coherence of the sentences nor SNR (0 *vs* −2 dB) affect the excitability of the lip motor cortex. These findings replicate the findings of Experiment 1. Furthermore, we found no evidence that the presence of noise modulates the excitability of the lip motor cortex, since no differences were found between clear sentences and sentences presented in noise. Moreover, excitability of the hand motor cortex was not modulated by listening to speech stimuli (relative to SCN and WN).

### 3.3 Analyses combining experiments 1 & 2

In order to compare experiments 1 and 2, we performed a 3-way ANOVA on the MEP z-scores with within-subject factor stimulus (coherent, anomalous & SCN) and the between-subjects factors TMS group (lip *vs* hand) and experiment (1 *vs* 2). The main effect of experiment was non-significant [F(1,50) = .17, *p* = .69], whereas the interaction between the TMS group, the stimuli and experiment showed a weak trend [F(2,100) = 2.69, *p* = .07]. A separate ANOVA for the hand group showed no significant main effects of stimulus and experiment, nor an interaction between these factors (all *p*-values greater than .11). The hand MEP z-scores (averaged across experiments) were increased relative to WN baseline for all stimulus types (one sample *t*-tests; coherent: *p* = .06; anomalous: *p* < .01; SCN: *p* < .05). A separate ANOVA for the lip group showed a strong main effect of stimulus [F(2,46) = 9.23, *p* < .001]. No main effect of experiment nor interaction involving experiment was detected (all *p*-values greater than .25). The MEP z-scores (averaged across experiments) were increased when listening to the anomalous and coherent sentences compared to the SCN (post-hoc Bonferroni pairwise comparisons: *p* < .01), but the semantic coherence had no effect on the MEP z-scores (post-hoc Bonferroni pairwise comparisons: *p* = 1). These results demonstrate that listening to sentences enhanced excitability of the lip motor cortex relative to SCN, but semantic coherence of the sentences did not modulate the excitability of the lip motor cortex. Listening to SCN also enhanced excitability of the lip motor cortex relative to the WN baseline (one sample *t*-test: *p* < .05).

These results show that there were no differences in the MEP z-scores between experiments 1 & 2. Listening to speech stimuli enhanced excitability relative to the SCN stimulus in the lip motor cortex, but not in the hand motor cortex.

## 4 Discussion

In this study, we aimed to address the hypothesis that the involvement of the articulatory motor cortex in speech processing increases when speech is difficult to understand. We manipulated the intelligibility and clarity of spoken sentences by modulating their SNR and semantic coherence. Results of Experiments 1 and 2 showed that listening to spoken sentences increased the excitability of the lip motor cortex more than listening to non-speech signals. Importantly, SNR and semantic coherence had no influence on the excitability of the lip motor cortex in either experiment. Thus, we found no supporting evidence for the hypothesis that the involvement of the articulatory motor cortex increases in challenging listening conditions. Our findings show that the articulatory motor cortex is involved in speech processing even in optimal and ecologically valid listening conditions and that its involvement is not modulated by the intelligibility and clarity of speech.

In both Experiments 1 and 2, listening to speech enhanced the excitability of the lip motor cortex relative to both non-speech signals, i.e., WN and SCN. This shows that the articulatory motor cortex is involved in speech processing in agreement with previous studies (Fadiga et al., 2002, Murakami et al., 2011, Watkins et al., 2003). The non-speech signals included in the current study had different temporal characteristics: WN is stationary, whereas SCN has a speech-like temporal structure. Listening to SCN enhanced excitability of both lip and hand motor cortex relative to WN. These enhancements of motor excitability were significant in the analyses combining data from experiments 1 and 2, although they were relatively weak and they did not reach significance in both experiments 1 and 2. Listening to speech also enhanced hand motor excitability, but it did not differ from the enhancement induced by SCN. In a recent study (Panouilleres & Möttönen, 2017), we found a similar pattern of results: listening to SCN and speech enhanced excitability in both articulatory and hand motor cortex relative to WN baseline. Listening to speech, however, caused a greater enhancement of excitability relative to SCN in the articulatory motor cortex, whereas no difference in excitability was found between speech and SCN in the hand motor cortex.

As pointed out above, the excitability of the hand motor cortex was slightly enhanced during listening to speech and non-speech signals with speech-like temporal structure (i.e., SCN) relative to WN. This suggest that the hand motor cortex is involved in processing of acoustic signals which have a rhythmic structure, including speech. It has been proposed that the motor cortex contributes to generation of temporal predictions, which affect perception of acoustic signals (Morillon and Baillet, 2017, Morillon et al., 2014). Temporal predictions may help to synchronize temporal fluctuations of attention with the stream of sensory events. The ability to focus attention on the most important features in continuous speech signals is likely to improve speech comprehension. Further research is needed to investigate the role of the motor cortex in controlling temporal attention during listening to speech and non-speech signals.

Our behavioural results showed that the semantically coherent sentences were more intelligible (Experiment 1) and clearer (Experiment 2) than semantically anomalous sentences when noise was added to sentences, replicating findings from previous studies (Davis et al., 2005, Miller and Isard, 1963). We hypothesized that if the articulatory motor system is involved in speech perception especially in challenging conditions (Wilson, 2009), the excitability should be enhanced more during listening to semantically anomalous sentences than semantically coherent sentences in noise. We found no support for this hypothesis as listening to coherent and anomalous sentences equally facilitated the excitability of the lip motor cortex relative to non-speech baselines.

We also manipulated the difficulty of speech perception by varying the SNR of the sentences. The SNR had a strong effect on intelligibility (Experiment 1) and perceived clarity (Experiment 2) of the spoken sentences. Despite this, no difference in excitability of the lip motor cortex was found between the five levels of SNR in Experiment 1 and between clear speech and speech in noise in Experiment 2. In contrast, two earlier studies have demonstrated an increase of lip motor excitability when passively listening to speech in noise relative to clear speech (Murakami et al., 2011, Nuttall et al., 2017). Nuttall et al. (2017) presented vowel-consonant-vowel stimuli during MEP recordings, whereas Murakami et al. (2011) presented sentences with and without white noise. Both Murakami et al. (2011) and Nuttall et al. (2017) repeated the same stimuli several times during their TMS experiments, whereas sentences were never repeated during the MEP recordings in the present study. Thus, differences in the type of noise (white noise versus Signal Correlated Noise), in the type of speech stimuli (sentences versus syllables) and in stimulus repetition could potentially explain the differences between the present results and previous ones. Nevertheless, our findings suggest that SNR of spoken sentences has no robust effect on the excitability of the articulatory motor cortex.

It is worth noting that in Experiment 2 that included clear speech stimuli the sample size was rather small in the lip group (*N* = 9), so one should be cautious when interpreting the lack of significant difference in excitability of the lip motor cortex between clear speech and speech in noise. We have recently run a larger study in which MEPs were recorded from the tongue muscle while 18 young adults listened to clear speech and speech in noise (SNR: 0 dB). No differences were found in the excitability of the tongue motor cortex in this study, in agreement with the findings of Experiment 2 (Panouilleres & Möttönen, 2017).

The present results highlight that the articulatory motor cortex is facilitated during passive listening to continuous and meaningful speech signals such as sentences. The majority of previous studies demonstrating the involvement of the articulatory motor system in speech processing have used syllables or single words as stimuli and often used identification and discrimination tasks (D'Ausilio et al., 2009, Du et al., 2014, Evans and Davis, 2015, Meister et al., 2007, Möttönen and Watkins, 2009, Pulvermüller et al., 2006, Smalle et al., 2015, Wilson et al., 2004). It is possible that these types of artificial stimuli and tasks activate cognitive processes that are not used in everyday speech communication. Indeed, it has been proposed that the motor activations may be related to these additional processes, not speech perception per se (Hickok & Poeppel, 2007). In agreement with previous studies (Murakami et al., 2011, Watkins et al., 2003), the present findings provide further evidence that the articulatory motor cortex is involved in processing ecologically valid speech signals, in the absence of behavioural tasks.

In the present study, the participants were instructed to listen to the sentences while MEPs were recorded. No tasks were included, because we aimed to make the listening conditions similar to everyday listening conditions. The previous studies demonstrating an effect of SNR on motor excitability also recorded MEPs during passive listening (Murakami et al., 2011, Nuttall et al., 2017). A possible confound of this design is that we did not control whether the participants actually payed attention to the sentences during the MEP recordings. It could be argued that the SNR and semantic coherence did not have an effect on the excitability of the articulatory motor cortex, because the participant did not attend to the sentences. Although we consider this to be unlikely, further studies are needed to examine how attention modulates motor excitability during listening to sentences. Our previous studies have shown that the articulatory motor cortex contributes to auditory processing of syllables even when they are unattended, but attention can further facilitate auditory–motor interactions (Möttönen et al., 2013, Möttönen et al., 2014b).

In the current TMS study, we measured changes of excitability in the motor cortex, but we did not manipulate it. Therefore, the study was not designed to test whether the articulatory motor cortex has a causal role in processing of sentences. Previous studies have however shown that TMS-induced modulation of motor areas influence performance in demanding speech discrimination tasks, in which syllables were presented in noise or close to the category boundary to increase task difficulty (D'Ausilio et al., 2009, Meister et al., 2007, Möttönen and Watkins, 2009, Smalle et al., 2015). Moreover, Schomers, Kirilina, Weigand, Bajbouj, and Pulvermüller (2015) showed that TMS over the tongue and lip motor representations in the left primary motor cortex affected reaction times in a word-to-picture matching task. Since the words were presented without noise in this study, these findings provide evidence that the articulatory motor system contributes to processing of meaningful speech in optimal listening conditions. Furthermore, TMS-induced disruptions in the articulatory motor cortex have been shown to modulate the processing of clear syllables in the auditory cortex (Möttönen et al., 2013, Möttönen et al., 2014b). These findings are in line with the present results and demonstrate that the articulatory motor regions play a causal role in processing clear speech as well as degraded speech. Future studies are needed to test whether the contribution of motor areas to speech perception is greater in challenging conditions than in optimal listening conditions. This is not a trivial question to address experimentally, because degrading speech sounds increases task difficulty and consequently sensitivity to measure effects of subtle motor manipulations on task performance (e.g., TMS-induced disruptions in the motor system, see Möttönen and Watkins, 2012, Schomers and Pulvermüller, 2016).

In conclusion, the present results show that processing of ecologically valid speech signals (i.e., spoken sentences) in the articulatory motor system is robust across a wide range of SNRs and across coherent and anomalous semantic context. This demonstrates that the articulatory motor system is involved in speech perception both in optimal and in challenging listening conditions.

## Appendix A Supplementary data

mmc1
