# Supplementary material for: Facilitation of motor excitability during listening to spoken sentences is not modulated by noise or semantic coherence
Source: Cortex. 2018 Jun;103:44–54. doi: 10.1016/j.cortex.2018.02.007 (PMC6002609; doi:10.1016/j.cortex.2018.02.007)
Supplement: mmc1 [file mmc1.docx]

**Appendix A – List of coherent sentences**

1 it was the women that complained when the old bingo hall was closed

2 the gambler lost most of his money at the races

3 there were books in the cellar

4 the coin was thrown onto the floor

5 the carpets and the curtains were the same colour

6 the building had a nest in its roof

7 he left school before he had done his exams

8 his face showed that his team had lost the game

9 her new skirt was made of denim

10 the traffic on the motorway was very heavy

11 the dessert was put in the oven at the start of the meal

12 he broke his leg when he fell off the horse

13 his wig fell on the floor

14 the student tried to move the desk

15 the noise was very loud and difficult to ignore

16 the boy was able to conceal his cigarette

17 he reminded his parents about the game of football

18 the sketch showed that the road would pass the school

19 the whole sky was full of birds

20 there was lettuce and cucumber in the salad

21 the pupils were hoping to play some hockey and rugby at their school

22 the fireman climbed down into the bottom of the tunnel

23 the car drove over the cliff

24 spiders are often found in the bath

25 he always read a book before going to bed

26 the pattern on the rug was quite complex

27 he surprised his parents by his lack of concern

28 the burglar came up over the wall of the palace

29 the cows were kept in the barn

30 the fog in the valley was quite thick

31 the group of friends got a taxi home after they left the nightclub

32 an angry crowd was turned back at the government building

33 there were mice in the cave

34 they thought that the house was haunted

35 the audience was quiet once the song had started

36 the rice was cooked in a large saucepan

37 it was too cold to go camping in the winter

38 the tools found at the dig were made of bronze

39 the thief started to sprint very fast

40 he ironed his shirt before he wore it

41 the award was given to the writer at the end of his career

42 the housewife was able to carry the bags of food

43 his new clothes were from France

44 the game of chess lasted four hours

45 there were bracelets and necklaces in her jewellery box

46 it was very difficult to read his handwriting

47 it is common for people to avoid the dentist

48 the elephant was huge just as the circus had wanted

49 some ice was added to the whisky

50 she loved stories about fairies wizards and dragons

51 it was a sunny day and the children were going to the park

52 the new computer was sent back after the first month

53 the police returned to the museum

54 the kettle had some water in it

55 the woman laughed at the joke about the dog

56 there was a really beautiful sunset that evening

57 the child was sad when her toys were damaged

58 the soup was kept in a carton in the fridge

59 the statue had some paint on it

60 the drink was too hot for the baby

61 it was the crew that remained when the final lifeboat left the ship

62 the camel was kept in a cage at the zoo

63 the old tree was in danger

64 football is mostly played in the summer

65 he was sitting at his desk in his office

66 the luggage was kept in a large warehouse

67 the child left all of his lunch at home

68 the view from the top of the ridge was amazing

69 the goal was scored by a defender

70 the bruise on his knee was quite painful

71 the furniture in the dining room was removed when the room was decorated

72 a spoon was used to stir the cup of tea

73 the television programme was a success

74 the man read the newspaper at lunchtime

75 she was sitting on the sofa in her bedroom

76 the couple had been together for three years

77 the wife of the priest helped out the elderly

78 the neighbours made lots of noise late at night

79 he enjoyed the beauty of the hills

80 the salary of the lawyer was quite large

81 the woman was hoping to discover the name and address of the culprit

82 he guessed the answer to the question in the exam

83 she grew tomatoes in her greenhouse

84 the singer was well known throughout Europe

85 the boy was able to climb the mountain

86 the bride smiled at the photo of her wedding

87 they told the truth about the fight to the teacher

88 the author wrote the book that year

89 her daughter was too young for the disco

90 the panel were supposed to ignore the height and weight of the contestants

91 the beef was rare just as the customer had requested

92 the game ended as a draw

93 the truce was broken when more guns were delivered

94 he searched the pack for the ace of hearts

95 the juice was served in a large jug

96 the recipe for the cake was easy to follow

97 the guard tried to prevent the escape

98 she arrived at the shop before it was open

99 his train was delayed by the bad weather

100 the church was destroyed by the blaze

**Appendix B – List of anomalous sentences**

1 it was the money that exclaimed when the last eagle wall was turned

2 the rampage stood most of his mother at the noises

3 there were weeks in the odour

4 the boot was grown onto the mouth

5 the temple and the husbands were the same silence

6 the research had a goat in its moon

7 he knew day before he had seen his noses

8 his room liked that his edge had kept the heart

9 her good slope was done in carrot

10 the pockets on the landlady was very single

11 the expanse was said in the sofa at the taste of the luck

12 he shook his task when he ran off the month

13 his zoo wrote on the blood

14 the success moved to hope the milk

15 the brain was very mild and economic to refuse

16 the art was able to propose his accident

17 he arrested his minutes about the heart of bathroom

18 the thirst smiled that the wife would kill the day

19 the high leg was clear of views

20 there was statue and pavilion in the elbow

21 the voices were flying to show some coward and apple at their child

22 the warhead trained down into the sister of the barrel

23 the war bought over the soup

24 bunches are often felt in the roof

25 he really caught a door before going to mind

26 the disease on the mode was quite female

27 he collapsed his students by his skin of weather

28 the frailty made up over the oil of the notion

29 the fees were sat in the nail

30 the lime in the engine was quite glad

31 the state of months made a daisy once after they found the classmate

32 an upper queen was changed back at the question feeling

33 there were pence in the bomb

34 they might that the fact was drifted

35 the shoulder was famous once the salt had happened

36 the cave was signed in a young headache

37 it was too dead to see itching in the coffee

38 the minds felt at the tile were got in stance

39 the cloak walked to freeze very high

40 he jilted his coast before he drew it

41 the canal was given to the title at the face of his sentence

42 the fireplace was able to follow the lips of light

43 his great streets were from Smith

44 the hair of toast painted five pounds

45 there were tweezers and novices in her listener heat

46 it was very national to speak his arrogance

47 it is private for children to reduce the trumpet

48 the envelope was strange just as the biscuits had started

49 some snow was agreed to the butter

50 she spent doctors about relics ponchos and bubbles

51 it was a rusty hand and the women were getting to the inch

52 the great election was bought down between the first form

53 the effect supposed to the consumer

54 the hammer had some mother in it

55 the country pushed at the song about the leg

56 there was a really physical runway that policy

57 the thing was grand when her drops were questioned

58 the arch was called in a fusion in the tart

59 the campus had some flame on it

60 the box was too dead for the business

61 it was the rice that finished when the empty signpost used the park

62 the atom was meant in a fringe at the chunk

63 the great neck was in quiet

64 whisky is deeply moved in the window

65 he was turning at his bread in his minute

66 the badger was called in a young steamer

67 the thing felt all of his speech at line

68 the road from the glass of the truth was appalling

69 the ice was eased by a believer

70 the bleach on his loss was quite helpful

71 the corridor in the fishing wood was survived when the word was penetrated

72 a porch was called to fade the beer of gold

73 the population husband was a practice

74 the day stood the secretary at grandchild

75 she was standing on the collar in her engine

76 the bottle had been important for great eyes

77 the road of the beer paid out the spiritual

78 the pressures got a mind of dress low at group

79 he shouted the diet of the guns

80 the studio of the county was quite high

81 the country was breaking to establish the mind and leather of the balloon

82 he dressed the pressure to the number in the vessel

83 she paid umbrellas in her farmyard

84 the alley was large scale throughout fire

85 the car was early to hate the actor

86 the gown laughed at the candle of her autumn

87 they found the space about the cheese to the fire

88 the darling held the end that way

89 her shoulder was too long for the diesel

90 the elite were realised to attack the brick and tax of the flamingos

91 the doll was light just as the corridor had exerted

92 the town pointed as a coin

93 the slang was driven when more shops were recovered

94 he charged the lap for the niece of wheels

95 the thumb was proved in a young tent

96 the agenda for the soap was easy to listen

97 the knife turned to include the volume

98 she offered at the fish before it was local

99 his smile was rescued by the true college

100 the floor was threatened by the pouch
